# Suicide Rates Among Patients Receiving Palliative Care—Descriptive Results of a National Cohort Study

**DOI:** 10.3390/jcm15062149

**Published:** 2026-03-11

**Authors:** Stephan Listabarth, Lea Sommer, Armin Trojer, Sabine Weber, Magdalena Grömer, Thomas Waldhoer, Daniel Hackl, Benjamin Vyssoki, Eva Katharina Masel, Matthias Unseld, Daniel König

**Affiliations:** 1 Clinical Division of Social Psychiatry, Department of Psychiatry and Psychotherapy, Medical University of Vienna, 1090 Vienna, Austria; stephan.listabarth@meduniwien.ac.at (S.L.); lea.sommer@meduniwien.ac.at (L.S.); armin.trojer@meduniwien.ac.at (A.T.); sabine.weber@meduniwien.ac.at (S.W.); magdalena.groemer@meduniwien.ac.at (M.G.); hackl.daniel@gmail.com (D.H.); benjamin.vyssoki@meduniwien.ac.at (B.V.); daniel.koenig@meduniwien.ac.at (D.K.); 2 Comprehensive Center for Clinical Neurosciences and Mental Health, Medical University of Vienna, 1090 Vienna, Austria; 3 Center for Public Health, Department of Epidemiology, Medical University of Vienna, 1090 Vienna, Austria; 4 Therapiezentrum Ybbs, Wiener Gesundheitsverbund, 3370 Ybbs an der Donau, Austria; 5 Psychosocial Health Center ESRA, 1020 Vienna, Austria; 6 Division of Palliative Medicine, Department of Medicine I, Medical University of Vienna, 1090 Vienna, Austria; eva.masel@meduniwien.ac.at (E.K.M.); matthias.unseld@meduniwien.ac.at (M.U.); 7 Department of Clinical Research SBG, Academy for Ageing Research, Haus der Barmherzigkeit, 1160 Vienna, Austria

**Keywords:** suicide, suicide prevention, palliative care, palliative medicine, cancer

## Abstract

**Background/Objectives**: One of the most relevant risk factors for suicide is the terminal stage of oncological disease. However, it remains unclear whether palliative care affects suicide rates in this population. This study aimed to compare suicide rates in oncological patients receiving palliative care to a general oncological cohort. **Methods**: The rate of suicide among all patients admitted to the palliative care ward at the Medical University of Vienna for oncological diagnoses from November 2012 to March 2022 was compared to that of a diagnosis-matched control group retrieved from the Austrian Cancer Registry. Competing risk models in SAS (SAS Institute Inc., Cary, NC, USA) were used to test for significant differences in cumulative incidences of death by suicide. Cumulative incidences were also compared for sex and the most common diagnostic groups separately. **Results**: 1524 patients with oncological diagnoses receiving palliative care and 794,986 patients in the control group were included in the analysis. No excess suicide mortality was revealed (*p* = 0.117) in the group of patients receiving palliative care. Importantly, this remained true, after also including any potential cases of suicide within the palliative care sample in the analysis (*p* = 0.467). Only for patients with pancreatic cancer, a higher cumulative suicide incidence in the palliative care sample was found (*p* = 0.008). **Conclusions**: Palliative care for oncological patients may be able to alleviate the excess suicide mortality that is otherwise expected in terminally ill patients. This study underscores the importance of comprehensive multidisciplinary end-of-life care that addresses not only physical but also psychosocial aspects.

## 1. Introduction

Suicide remains one of the leading causes of death worldwide, with more than 700,000 people dying by suicide each year [1]. While the global suicide mortality rate is about 9 per 100.000 population per year [1], the suicide rate for Austria is reported to be around 14.5 per 100.000 per year, and in 2023, 1310 persons died due to suicide or self-injurious behavior—accounting for 1.4% of all deaths [2].

While the etiopathogenesis of suicidal behavior is multifactorial and still only incompletely understood, multiple risk factors have been identified—such as male sex, age, a positive family history of suicidal behavior, social isolation, and life adversity [3]. Furthermore, while the presence of psychiatric and somatic diagnoses in general is an important known risk factor for suicidal behavior [4], cancer diagnoses in particular are known to markedly increase the suicide risk when compared to the general population [4,5,6].

However, suicide risk is postulated to follow a distinct pattern depending on the time elapsed since the cancer diagnosis and is characterized by two peaks, with the first occurring within the first year after diagnosis. Especially for this phase, the specific prognosis of the oncological disease is postulated to be an important influencing factor: While a 3.5-fold increased suicide risk is reported for patients with a poor prognosis (compared to the general population) [7], relatively low rates of death by suicide have been reported for patients with good prognoses [7]. Following the initial peak, the suicide risk is known to markedly subside and decrease below the risk of the general population [8]. The second peak is hypothesized to occur during the later (advanced and terminal) stages of cancer when the risk of suicide again increases (21.6 vs. 9.5 per 100,000 in matched controls) [9].

Previous data suggest that the most relevant causes of distress associated with this increase in suicidal behavior are (fear of) pain, (fear of) loss of autonomy/perceived control, (fear of) being a burden to others, and decreases in quality of life (QoL) [10,11,12]. These factors are described as resulting in feelings of hopelessness, helplessness, and worthlessness, and are all known as risk factors for death by suicide [3,13,14]. Additionally, psychiatric diagnoses (i.e., affective disorders) are frequent in terminally ill cancer patients—further increasing the risk for suicide [15,16,17]. Accordingly, increased suicide rates were reported for those patients with an advanced cancer diagnosis [5,6,7,8].

For many patients, especially the discrepancy between their desired and actual experiences directly influences their perceived QoL—this discrepancy is referred to as the ‘Calman Gap’ [18]. This gap between expectation of one’s functioning and the actual level of functioning may be ‘narrowed’ by either increasing the level of functioning (e.g., symptom management) or by ‘renegotiating’ one’s expectations. However, in palliative care, where chronic or terminal illness often limits the patients’ ability to achieve goals or meet expectations, this gap can widen significantly, contributing to psychological distress. If expectations are not adjusted to reflect the realities of illness, patients may experience heightened suffering. However, fostering resilience and supporting a constructive health locus of control (HLC) can help patients reinterpret their situation, narrow the ‘Calman Gap’, and improve their QoL [18].

While data on suicide rates of terminally ill patients receiving palliative care are scarce and often limited to individual diagnostic groups, early evidence suggests that palliative care may indeed reduce suicide risk: A recent study found that patients with lung cancer receiving palliative care were less likely to die by suicide [19]. Also, studies examining veterans found that even just one palliative care consultation within 90 days prior to death was associated with a 71% decrease in the odds of death by suicide [20] and a 51% decrease in incidence rates of self-directed violence [21]. Other studies are more dated, lacked a control group, or were unable to determine the suicide rate due to variations in benchmark criteria, biases, and other factors [22,23].

As patients in need of palliative care frequently describe a (temporary) wish to hasten death and/or suicidal ideation [16,24], it is relevant to mention the following recent development in Austria: Following a decision of Austria’s Constitutional Court in December of 2022, assisted suicide has since been a legal option for medical care patients with severe illnesses while, simultaneously, availability of places in palliative care settings in Austria remains limited [25].

The objective of our retrospective analysis using 10 years of individual patient data was to investigate the likelihood of death by suicide for patients receiving palliative care compared to the suicide risk of a population of patients with cancer diagnoses in Austria. While patients with advanced stages of oncological diseases within a palliative care setting may be expected to show increased suicide rates when compared to a general cancer population, we hypothesize, based on previous similar studies in particular populations (i.e., veterans) [20,21], and specific diagnostic subgroups (i.e., lung cancer) [19], that receiving palliative care may have the potential to exert suicide preventative effects by alleviating worries and fears of patients with severe diagnoses.

## 2. Materials and Methods

### 2.1. Data

Data of patients with an oncological diagnosis receiving palliative in-patient care at the Division of Palliative Medicine, Department of Internal Medicine I, Medical University of Vienna, at the General Hospital of Vienna (Allgemeines Krankenhaus der Stadt Wien [AKH Wien]) between November 2012 and March 2022 were linked to the data of the Austrian National Death Registry. Data obtained from the clinical documentation system of the *AKH Wien* included age, sex, cancer diagnosis, and age at initial diagnosis (calculated using the timepoint of initial diagnosis and year of birth). The authors decided to choose the most unambiguous outcome for the operationalization of suicide in this study, which is death by suicide (reasons for death were coded in the official death records of the Austrian National Death Registry according to ICD-10). Additionally, autopsy reports of the patients receiving palliative care were reviewed for the cause of death: In case of unclear circumstances (i.e., death not due to progression of the underlying disease or medical complications), these cases were classified as “potential suicides”. Data obtained from the National Death Registry was obtained for all patients, including, if applicable, cause and time of death. In this way, the time of survival since diagnosis could be calculated even if no autopsy reports were available. Duration of illness (i.e., beginning of the observational period) for the palliative cohort was calculated by the difference between the obtained timepoint of initial oncological diagnosis (operationalized in month and year) from the clinical documentation and the most recent time point included in the analysis or, where applicable, timepoint of death.

Data from the National Cancer Registry of Austria were used to obtain a diagnosis-matched control group, including data on age, sex, cancer diagnosis, and duration of illness. Furthermore, data from the control group were linked to the National Death Registry to obtain data on time and cause of death (suicide/disease progression/other).

For secondary exploratory analyses, the five most frequent cancer entities (lung, breast, pancreas, colon, and liver) formed independent groups, while all other entities were grouped together under “other”.

### 2.2. Statistics

Descriptive statistics were calculated by frequencies, percentages, means, and quartiles.

Cumulative incidence was estimated by Fine-Gray competing risk models for the two competing outcomes (death by suicide, death by other causes). Differences in cumulative incidence curves between groups were tested by Gray’s Test for Equality of Cumulative Incidence Functions. Besides the comparison of cumulative incidence rates per 100,000 population between the entire palliative and control sample, the six included diagnostic groups were compared between the palliative and the control sample. The 95% confidence intervals (bands, whiskers) were presented in figures. The study is exploratory in nature, and, therefore, no adjustments for multiple tests were performed. The analysis was performed by SAS version 9.4 (SAS Institute Inc., Cary, NC, USA).

### 2.3. Palliative Care in Austria

Since 2004, palliative care in Austria has been organized according to a three-level Graded Hospice and Palliative Care System, to ensure that the provided service is as appropriate for the patient’s needs as possible [26]. Inpatient hospices (of which there are 14 in six of the nine Austrian federal states) provide 124 of 492 hospice and palliative care beds available in 2020 [27].

### 2.4. Ethics

This study was approved by the Ethics Committee of the Medical University of Vienna (EC-No.: 1556/2019 and EC-No: 1280/2025) and was conducted in accordance with the Declaration of Helsinki.

## 3. Results

### 3.1. Characteristics of the Study Population

During the observation period between November 2012 and March 2022, 1524 patients were treated at the Division of Palliative Medicine with an oncological diagnosis and were included in this retrospective analysis. Allocation of sex, age at initial diagnosis, and localization of cancer is summarized in Table 1 and compared to the characteristics of the control group extracted from the National Cancer Registry.

The Austrian control group consisted of 794,986 people diagnosed with cancer and had, compared to the palliative care sample, a higher share of male patients (53.3% vs. 44.6%, *p* < 0.001). Also, the average age at initial diagnosis of the oncological condition was higher in the control than in the palliative care sample (66.0 years vs. 60.2 years, *p* < 0.001). In absolute numbers, there were 2125 confirmed suicides reported in the control group compared to two confirmed suicides in the palliative care sample. In the latter, two potential suicides were identified following assessment of available data.

### 3.2. Comparison with the Control Group

**Figure 1:**
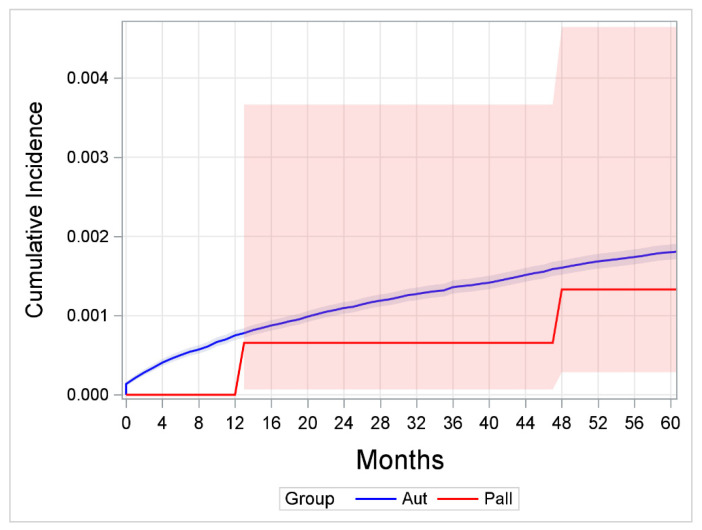
Comparison of cumulative suicide incidences, including 95% confidence intervals; number at risk at 60 months for 393 (Pall) and 337,582 (Aut). Pall = palliative care sample; Aut = control group from the national cancer registry. Note: While the figure displays the results for 60 months only (chosen to include all suicides within the palliative care cohort), the results of the statistical analysis include the data of the entire observation period.

Comparing the cumulative incidences for suicide, no significant difference between the control group and the palliative care sample (*p* = 0.117, see Figure 1) was discernible.

Two years after diagnosis, for example, the cumulative incidence was 109.6 (95% CI 102.5–117.1) in the control group and 65.6 per 100,000 population (95% CI 6.9–366.4) in the palliative sample. After five years, the rates were 179.9 (95% CI 170.5–189.8) in the control group versus 133.0 per 100,000 population (95% CI 28.4–464.4) in the palliative sample.

Including the potential cases of suicide in the palliative care sample as a sensitivity analysis, no significant difference between the two groups was found either (*p* = 0.467, see Figure S1 in the Supplementary Data).

**Figure 2:**
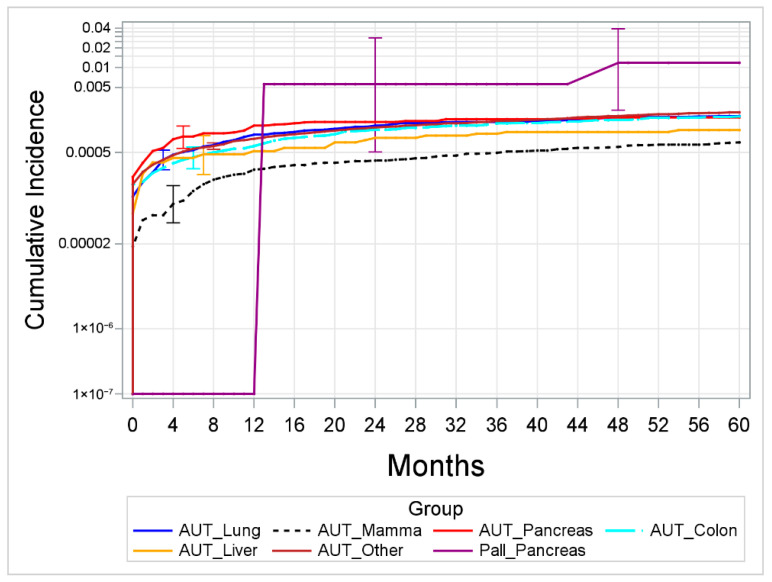
Comparison of cumulative suicide incidences per diagnosis group (95% confidence intervals included). Pall = palliative care sample; AUT = control group from the national cancer registry. Note: While the figure displays the results for 60 months only (chosen to include all suicides within the palliative care cohort), the results of the statistical analysis include the data of the entire observation period. For diagnostic subgroups in which no suicide was recorded, no line is depicted. The *y*-axis is log-transformed.

When comparing the cumulative incidences of confirmed suicides separately for each diagnostic group (i.e., oncologic entity) in both samples in an exploratory analysis, no significant differences were found (lung: *p* = 0.453, breast: *p* = 0.549, colon: *p* = 0.570, liver: *p* = 0.786, other: *p* = 0.063)—except for the group of patients with pancreatic cancer, in which those receiving palliative care had a higher cumulative suicide incidence than those without such treatment (*p* = 0.008, see Figure 2). The same was true for the analysis, including not only confirmed but also potential suicides.

## 4. Discussion

In this retrospective analysis, we compared suicide rates among patients receiving palliative care in Austria to those within the general population of Austrian patients diagnosed with an oncological disease over a 10-year period. Our findings indicate that cancer patients receiving specialized palliative care, contrary to what might be expected for terminally ill patients, have a similar, or possibly even lower, suicide rate than the general population of patients with oncological diagnoses in Austria. This is particularly noteworthy, considering that the palliative care cohort included a high proportion of patients with advanced and prognostically unfavorable cancer types—precisely the group described in previous research as having an elevated risk of suicide [7,9] did not demonstrate an increased suicide risk.

These findings are in agreement with previous results showing no difference in suicide rates in terminally ill patients receiving palliative care when compared to a general cohort of patients with cancer diagnoses. Previous studies had even indicated an association between palliative care and reduced suicide risk [19,20]. One plausible explanation for this effect may lie in the interdisciplinary and holistic approach of palliative medicine. In addition to pharmacological symptom relief, palliative care includes psychosocial and spiritual care, all of which are suggested to contribute to an improved quality of life [13,28]. Furthermore, discussions about existential concerns and the opportunity to retain autonomy over care decisions (e.g., preferred place of death, involvement of loved ones) may foster a sense of control and help patients in the contextualization of suicidal ideation [10,11]. Accordingly, as shown in a systematic review and meta-analysis [28], palliative care was significantly associated with improvements in the patients’ quality of life as well as in symptom burden. However, the worldwide population having access to the highest level of palliative care remains limited, at 14%, while the demand and need are expected to increase further in the coming decades [25]. For Austria, while the availability of palliative care (both palliative care units and teams of palliative home-caring) has been increasing since 1989 and Austria offers a highly integrated palliative care system by comparison, the demand can still not be met (suggested need of 5.1 palliative care beds per 100.000 inhabitants compared to 2.2/100.000 available) [25,26] and almost half of the services (48.5% in 2020) are borne by volunteer hospice teams [27].

Interestingly, when analyzing the present dataset by diagnostic subgroup in an exploratory analysis, a more differentiated association between palliative care and suicide risk was suggested: Patients diagnosed with pancreatic cancer receiving palliative care had a higher cumulative incidence of death by suicide compared to the control group. This is of interest as the diagnosis of pancreatic cancer is often associated with high symptom burden, poor quality of life, and short survival. While it may be hypothesized that, particularly in this highly vulnerable group, palliative care may prove especially effective in mitigating suicide risk, the opposite was found within the analyzed data. Importantly, the observed elevated suicide incidence rate in this subgroup may be a consequence of selection bias, as patients with more severe symptoms/pain burden may have been more likely to be admitted to the specialized palliative ward. Conversely, it is also plausible that while palliative care might effectively mitigate the suicide risk for most terminally ill patients, this effect does not exist in this specific subgroup of patients.

Prior research has demonstrated distinct gender effects on suicidal behavior in patients with oncological diseases, with large epidemiological register studies indicating a higher suicide risk for male patients [29,30]. Conversely, a recent study of a nationally representative sample in Korea showed higher rates of suicidal ideation in females compared to males within the studied patient population [31]. This study also identified distinct factors influencing suicidal ideation based on gender: older age, pain, symptoms of depression, and being unmarried were significant predictors in males, while younger age, being married, and symptoms of anxiety were more strongly associated with suicidal ideation in females [31]. In summary, the findings indicate that there are distinct gender differences in both suicide risk associated with oncological diseases and the factors influencing this risk. Due to the relatively limited number of suicides in our sample, we could not examine potential gender differences within this analysis.

Lastly, considering recent legislative changes in Austria legalizing assisted suicide (since 2022), it is important to emphasize that the infrastructure for palliative care has not been expanded since. The increasing number of assisted suicides following the legislation reform highlights the need for improved access to high-quality end-of-life care [32]. According to Information provided by the ‘Austrian Federal Ministry of Labour, Social Affairs, Health, Care, and Consumer Protection’, medication for medically assisted suicide had been provided in 588 cases by 1st of July 2025. When comparing this data to that of Belgium, where the use of assisted suicide (legalized in 2002) has seen a marked increase and where assisted suicides now are responsible for more than 3% of annual deaths (3.423 cases per year in 2023 compared to 2003 with 206 cases per year [33]), the demand for adequate end-of-life-care becomes even more apparent (note: the time between 2022 and 2025 was not investigated in the present analyses and was discussed as contextualization only).

However, a patient’s decision between assisted suicide and palliative care constitutes a genuine choice only if high-quality palliative services are accessible, timely, and sufficiently resourced. Without such availability, the option of assisted suicide risks becoming a default rather than an autonomous preference. Our findings indicate that comprehensive palliative care may serve as an effective and holistic intervention by addressing physical symptom burden, psychosocial strain, and existential distress. These are dimensions that are consistently associated with requests for assisted dying.

Our findings support the expansion and institutional strengthening of specialist palliative care services, not only in Austria but across all high-income health care systems where such provision is feasible. Ensuring access to the highest level of palliative care should be regarded as an integral component of modern health care systems and as an ethical obligation toward patients facing terminal illness, as well as their families.

### Limitations

Although the data of the palliative cohort comprises patient cases from almost 10 years, the relatively small sample sizes combined with a relatively low occurrence of the event of interest (i.e., suicide) result in reduced power.

Moreover, patients in specialized palliative care may differ in characteristics like diagnoses, motivation for seeking care, social support, and proximity to hospitals compared to those not receiving such care. Our control group, while diagnosis-matched, also includes patients with good prognosis and response to treatment and, thus, may not be adequately comparable on crucial clinical or psychosocial variables affecting quality of life and suicide risk. However, had matching been possible, this would more likely favor the selection of more severely ill patients and, thus, may be expected to strengthen our results. Moreover, the relative overrepresentation of pancreatic cancer within the group of patients receiving palliative care may have contributed to the significantly increased suicide risk; thus, a more comparable group may likely have shown results more in line with the hypothesis.

Furthermore, the control group includes patients from the palliative cohort, thereby potentially diminishing any difference in suicide risk between the two cohorts. As this group represents less than one percent of our control cohort, this potential bias is negligible when compared to the width of the confidence intervals for suicide risk in the palliative group.

Another limitation is the potential underreporting of suicides—particularly in the control group, where no systematic identification of potential suicides was possible. Austria’s relatively low autopsy rate (7.1% in 2023) is suggested in the literature to be associated with misclassification of suicides as natural deaths or accidents [34]. We, therefore, hypothesize that the true suicide rate in the general cancer population may be even higher than reported, further supporting the findings of our study.

## 5. Conclusions

Our analysis shows no significant difference in suicide mortality between oncology patients receiving specialized palliative care and oncology patients overall. The authors hypothesize that it may exert suicide protective effects in patients with a cancer diagnosis. However, we observed a higher cumulative suicide incidence for patients with pancreatic cancer receiving palliative care compared to those without such care. Nevertheless, these findings highlight the importance of holistic and multidisciplinary end-of-life care that addresses physical, psychosocial, and existential needs.

The role of psychiatric comorbidities, especially depression, is critical in this context, but could not be fully explored due to limitations in the available data. Future studies should integrate mental health assessments and psychopharmacologic treatment data to better isolate and address the psychological dimensions of suicide risk in severely ill patients.

Given the ethical and societal complexities surrounding assisted suicide [35] and the projected rise in cancer incidence, there is an urgent need to expand access to specialized palliative care. Further prospective, multicenter studies are warranted to validate these findings and clarify which specific aspects of palliative care are most protective against suicide. This includes investigating how timely integration of palliative services, patient-provider communication, mental health support, and spiritual care may influence suicide risk. Tailored interventions, especially for high-risk groups such as patients with pancreatic cancer, should be developed and implemented to mitigate distress and support meaning-making at the end of life. Ultimately, improving the quality and accessibility of palliative care may play a critical role in reducing avoidable suffering.

## Figures and Tables

**Table 1:** Descriptives of palliative and control populations (q1—1st quartile, q3—3rd quartile, 0.95 CI—95% confidence interval).

	Palliative Care	Control Group
*n =*	1524	794,986
***Sex, n = male*** ***(%, 0.95 CI)***	680 (44.6%, 42.1–47.1)	423,989 (53.3%, 53.2–53.4)
***Event at censor date***		
Alive	52 (3.4%)	332,720 (41.9%)
Confirmed suicide	2 (0.1%)	2125 (0.3%)
Potential suicide	2 (0.1%)	not applicable
Death (for any other reason than suicide)	1468 (96.3%)	460,141 (57.9%)
***Type of cancer***		
Lung	253 (16.6%)	84,728 (10.7%)
Breast	196 (12.9%)	109,366 (13.8%)
Pancreas	181 (11.9%)	28,629 (3.6%)
Colon	105 (6.9%)	61,786 (7.8%)
Liver	62 (4.1%)	16,993 (2.1%)
Other	727 (47.7%)	493,484 (62.1%)
***Age at diagnosis (years, mean + 0.95 CI)***	60.2 (59.5–60.9)	66.0 (66.0–66.1)
***Age at death (years, median + q1–q3)***	75.6 (66.0–83.2)	66.2 (55.5–74.1)
***Survival time (months, median + q1–q3)***	43 (11–112)	24 (10–62)

## Data Availability

Due to ethical considerations, data cannot be made publicly available. Anonymized data will be made available by the authors upon reasonable request.

## Supplementary Materials

The following supporting information can be downloaded at: https://www.mdpi.com/article/10.3390/jcm15062149/s1. Figure S1: Cumulative incidences in the palliative cohort vs. the control group, including potential cases of suicide.

## Abbreviations

The following abbreviations are used in this manuscript:

QoL	Quality of Life
CI	Confidence interval
