# Supplementary material for: Suicide Rates Among Patients Receiving Palliative Care—Descriptive Results of a National Cohort Study
Source: J Clin Med. 2026 Mar 11;15(6):2149. doi: 10.3390/jcm15062149 (PMC13026687; doi:10.3390/jcm15062149)
Supplement: Supplementary file 1 [file jcm-15-02149-s001.zip › jcm-4133821-supplementary.pdf]

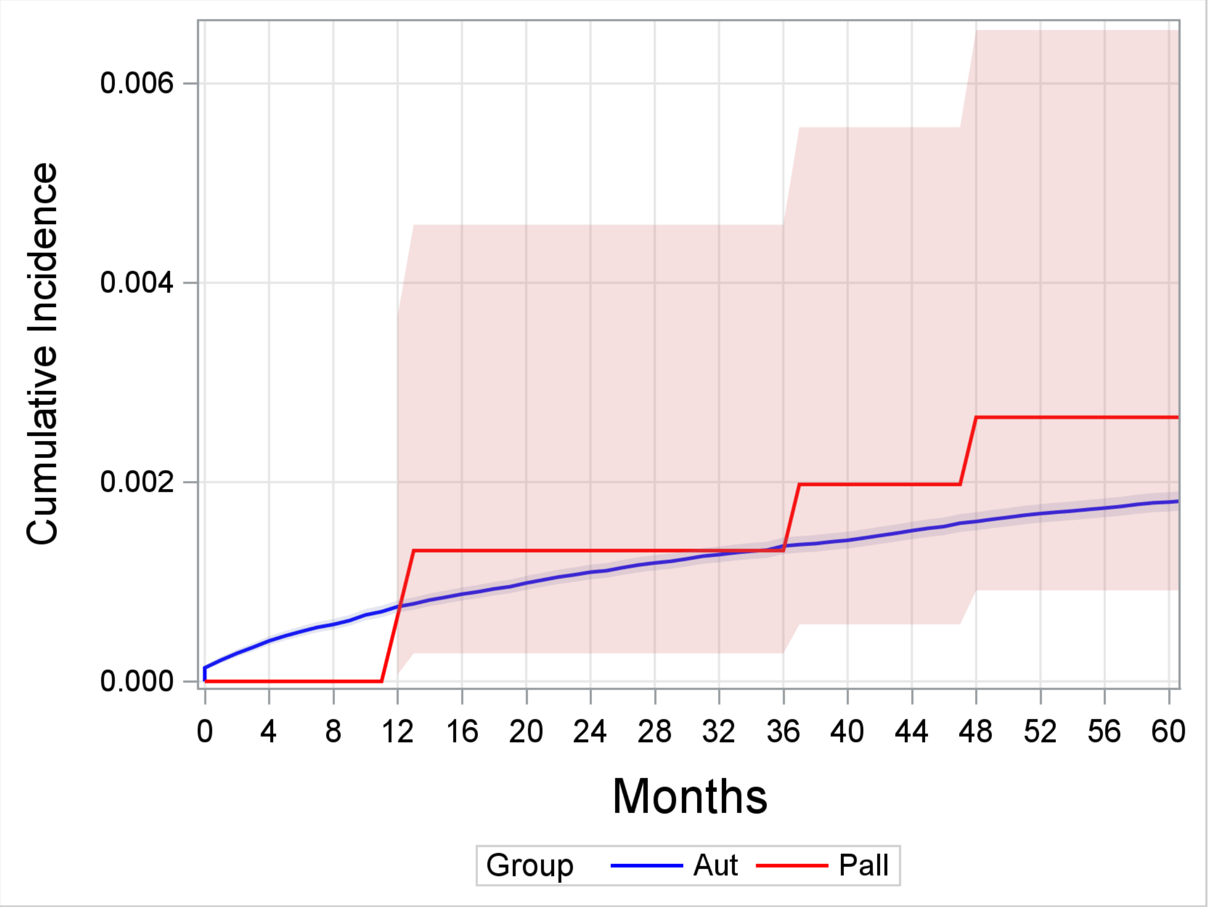


*Figure S1: Comparison of cumulative suicide incidences based on potential and confirmed suicides cases including 95% confidence intervals, Pall = palliative care sample, Aut = control group from national cancer registry*
